# Beneficial immunostimulatory effect of short-term *Chlorella* supplementation: enhancement of *Natural Killer* cell activity and early inflammatory response (Randomized, double-blinded, placebo-controlled trial)

**DOI:** 10.1186/1475-2891-11-53

**Published:** 2012-07-31

**Authors:** Jung Hyun Kwak, Seung Han Baek, Yongje Woo, Jae Kab Han, Byung Gon Kim, Oh Yoen Kim, Jong Ho Lee

**Affiliations:** 1 Yonsei University Research Institute of Science for Aging, Yonsei University, Seoul, Korea; 2 Department of Biology, College of Life Science and Biotechnology, Yonsei University, Seoul, Korea; 3 Interdisplinary course of Science for Aging, Graduate School, Yonsei University, Seoul, Korea; 4 Daesang.Co.Ltd, Kyunggi-do, Korea; 5 Department of Food Science and Nutrition College of Human Ecology, Dong-A University, Busan, Korea; 6 Department of Food & Nutrition, College of Human Ecology, Yonsei University, 134 Shinchon-dong, Sudaemun-Gu, Seoul, 120-749, Korea

**Keywords:** Chlorella, *Natural Killer* cell activity, Interleukin-12, Interferon-γ, Interleukin-1β, Immunostimulatory effect

## Abstract

**Background:**

*In vitro* and animal studies have demonstrated that *Chlorella* is a potent biological response modifier on immunity. However, there were no direct evidences for the effect of *Chlorella* supplementation on immune/inflammation response in healthy humans.

**Methods:**

This study was designed for an 8-week randomized, double-blinded, placebo-controlled trial: 5g of *Chlorella* (n=23) or Placebo (n=28) as form of tablets. Mainly, cytotoxic activities of *Natural killer (NK)* cells and serum concentrations of interferon-γ, interleukin-1β and interleukin-12 were measured.

**Results:**

After the 8-week, serum concentrations of interferon-γ (p<0.05) and interleukin-1β (p<0.001) significantly increased and that of interleukin-12 (p<0.1) tended to increase in the *Chlorella* group. The increments of these cytokines after the intervention were significantly bigger in the Chlorella group than those in the placebo group. In addition, *NK* cell activities (%) were significantly increased in *Chlorella* group, but not in Placebo group. The increments of *NK* cell activities (%) were also significantly bigger in the *Chlorella* group than the placebo group. Additionally, changed levels of *NK* cell activity were positively correlated with those of serum interleukin-1β (r=0.280, p=0.047) and interferon-γ (r=0.271, p<0.005). Signficantly positive correlations were also observed among the changed levels of serum cytokines; between interferon-γ and interleukin-1β (r=0.448, p<0.001), between interleukin-12 and interleukin-1β (r=0.416, p=0.003) and between interleukin-12 and interferon-γ (r=0.570, p<001).

**Conclusion:**

These results may suggest a beneficial immunostimulatory effect of short-term *Chlorella* supplementation which enhances the *NK* cell activity and produces interferon-γ and interleukin-12 as well as interleukin-1β, the Th-1 cell-induced cytokines in healthy people.

## Background

*Chlorella* is an unicellular green algae which contains essential amino acids, protein, minerals, vitamins, dietary fiber, and a wide range of antioxidants, bioactive substances and chlorophylls etc. [1,2]. It has been a popular foodstuff worldwide, especially in Asian countries i.e. Japan, Taiwan as well as Korea.

Various pharmacological effects of *Chlorella* have been expressed not only in animal models [3,4] but also in human experiments [5-7]. According to Okudo et al [5], the blood cholesterol levels were reduced in hypercholesterolemic patients who consumed *Chlorella*. Fujiwara et al [6] also showed a beneficial effect of *Chlorella* consumption on hyperlipemia. Nakamura et al demonstrated that *Chlorella* intakes reduced blood pressure in mildly hypertensive people [7]. *Chlorella* supplementation for 6-week, brought a favorable impact on antioxidant status in male smokers [8]. In addition, *Chlorella* showed a considerable antioxidant effect and a reduction of blood glucose levels in diabetic animal models and stress-induced ulcer mice [9-15].

In fact, *in vitro* and animal studies have demonstrated that *Chlorella* or *Chlorella* extract are involved in the modulation of immune responses against tumors [16-18], bacterial and viral infection [19-23] as well as native Ags i.e. casein [24]. It has been reported that oral administration of *Chlrorella* extract enhanced the resistance to infection with *Listeria monocytogenes*, an intracellular bacterium, through the augmentation of T-helper-1 (Th1) cell response both in normal and immunocompromised hosts [21,22]. However, there were no direct evidences on the effect of *Chlorella* supplementation on immune response in humans, particularly in uninfected normal people. Therefore, this study aimed to investigate if *Chlorella* supplementation gives a beneficial immunostimulatory effect to uninfected normal people.

## Materials and methods

### Study participants

Study subjects were recruited from the Health Service Center of Yonsei Hospital. Subjects were excluded if they have any history of the following: 1) clinical or electrocardiographic evidence of coronary artery disease, stroke, myocardial infarction, or peripheral arterial occlusive disease; 2) diabetes mellitus (fasting glucoses ≥126mg/dL or 2hr serum glucose ≥200mg/dl after a 75g oral glucose tolerance test); 3) abnormal liver or renal function; 4) thyroid or pituitary disease; 5) acute or chronic inflammatory/immune disease including malignant tumor, lung disease, leukemia, autoimmune disease 6) lactose intolerance, 7) orthopedic limitations; 8) body weight loss/gain≥10% in the past 1 year; 9) regular use of any medications that could affect cardiovascular function and/or metabolism; and 10) the number of white blood cells >8 × 10^3^/uL. The aim of the study was carefully explained to the volunteers, their written informed consent was obtained and the protocol was approved by the Ethics Committee of Yonsei University. Finally, 60 people were enrolled in this study.

### Study design and *Chlorella* source

This study was designed for an 8-week randomized, double-blinded, placebo-controlled trial. Sixty subjects were randomly assigned to receive placebo (n=30) or 5g of *Chlorella* (n=30) as form of tablets. Test-product pills contained only dried *Chlorella* (97% purity) extracted from 100% of *Chlorella vulgaris* (*CVE*) (Daesang Corp., Seoul, Korea) as an active ingredient and placebo-product pills contained lactose in identical-looking tablets. Subjects were instructed to consume 5 g/d of *Chlorella* (12 pills/d) or placebo (12 pills/d) by taking 4 pills after each main meal. All participants were encouraged to maintain their usual lifestyle and dietary habits. Compliance was assessed by counting the remained tablets and food records. If the tablets are consumed more than 80%, compliance was considered good.

### Anthropometric parameters and blood collection

Body weight and height were measured unclothed and without shoes in the morning. Body mass index (BMI) was calculated as body weight in kilograms divided by height in square meters (kg/m^2^). Blood presusure (BP) was obtained from the left arm of seated patients with an automatic blood pressure monitor (TM-2654, A&D, Tokyo, Japan) after 20 min of rest. After overnight fast (12-hours), venous blood specimens were collected in EDTA-treated and plain tubes. Tubes were immediately placed on ice until they arrived at the analytical laboratory (within 1-3h). Blood specimens in EDTA tubes were used for the isolation of peripheral blood mononuclear cells (PBMC) and those in plain tubes were separated into serum and stored at -70°C until analysis.

### Isolation of peripheral blood mononuclear cells (PBMC)

Whole blood was mixed with the same volume of RPMI 1640 (Gibco, Invitrogen Co, USA) and gently laid on a histipaque-1077(Sigma, CA, USA), then centrifuged at condition in 2000rpm, 20 min, 10°C. After the separation, a thin layer of PBMC coat indicating PBMC was isolated, washed twice with RPMI 1640 and resuspended in RPMI 1640 with streptomycin. They were used for *Natural killer (NK)* cell cytotoxicity assay.

### Cytotoxic activities of *Natural killer (NK)* cells

The cytolytic activities of *NK* cells were determined by CytoTox 96® Non-Radioactive Cytotoxicity Assay Kit (Promega Co., WI, USA). For *NK* cell cytotoxic activity, PBMCs isolated from each subject were incubated with K562 cells. Briefly, PBMC cells (effector cell, E) were seeded in the well in a ratio of 5:1 and 1.25:1 with the K562 cells (2×10^4^cells/well) (targeted cell, T). The plates treated at different ratio of E:T (5:1 and 1.25:1) were incubated at 37°C with 5% CO_2_ for overnight according to the manufacturer's instructions. Finally, *NK* cell activity of effector cells was measured with 2030 multilable reader (Victor^™^ x5, PerkinElmer, USA) at 490nm and was calculated with this formula.

(1) $$\%\;\text{Cytotoxicity}=\frac{\text{Experimental}-\text{Effector Spontaneous}-\text{Target Spontaneous}}{\text{Target Maximum}-\text{Target Spontaneous}}\times 100$$

### Cytokine assay for serum concentrations of interferon-γ, interleukin-1β and interleukin-12

Serum concentrations of interferon-γ (IFN-γ), interleukin-1β (IL-1β) and interleukin-12 (IL-12) were measured using Bio-Plex Pro^™^ Assay kit (Bio-Rad Laboratories, Inc., Hercules, CA, USA) according to the manufacturer's instructions.

### Serum lipid profile and white blood cell count

Fasting serum levels of total cholesterol and TG were measured using commercially available kits on a Hitachi 7150 Autoanalyzer (Hitachi Ltd. Tokyo, Japan). After precipitation of serum chylomicron, low density lipoprotein (LDL) and very low density lipoprotein (VLDL) with dextran sulfate-magnesium, HDL cholesterol (HDL-C) left in the supernatant was measured by an enzymatic method. LDL cholesterol (LDL-C) was estimated indirectly using the Friedewald formula for subjects with serum TG concentrations <400 mg/dL (4.52 mol/L). In subjects with serum TG concentrations ≥400 mg/dL (4.52 mol/L), LDL-C was measured by an enzymetic method on a Hitachi 7150 Autoanalyzer directly. White blood cell (WBC) count was determined using the HORIBA ABX diagnostic (HORIBA ABX SAS, ParcEuromedicine, France).

### The assessment of dietary intake and physical activity level

The subjects' usual diet information was obtained using both a 24-hour recall method and a semi-quantitative food frequency questionnaire (SQFFQ) of which the validity had been previously tested [25]. We used the former to carry out analyses and the latter to check if the data collected by 24-hour recall methods was representative of the usual dietary pattern. All the subjects were given written and verbal instructions by a registered dietitian on completion of a 3-day (2 week days and 1 weekend) dietary record every 4 weeks. Dietary energy values and nutrient content from a 3-day food records were calculated using the Computer Aided Nutritional analysis program (CAN-pro 2.0, Korean Nutrition Society, Seoul, Korea). Total energy expenditure (TEE) (kcal/day) was calculated from activity patterns including basal metabolic rate, physical activity for 24 hours [26], and specific dynamic action of food. Basal metabolic rate for each subject was calculated with the Harris-Benedict equation [27].

### Statistical analysis

Statistical analyses were performed using SPSS version 15.0 for Windows (Statistical Package for the Social Sciences, SPSS Inc., Chicago, IL, USA). For intra-group tests, we conducted paired *t*-tests. For inter-group comparison, student *t* -test was used to compare initial value or absolute (net) differences. Frequencies were tested by chi-square test among groups. Pearson correlation coefficients were used to examine relationships between variables. We determined whether each variable was normally distributed before statistical testing, and logarithmic transformation was performed on skewed variables. For descriptive purposes, mean values are presented using untransformed values. Results are expressed as mean±S.E or %. A two-tailed value of p<0.05 was considered statistically significant.

## Results

Among the enrolled subjects (n=60), 9 subjects dropped out and 51 subjects completed the study. Among the 9 drop-outs, 7 were in the placebo group (2 for personal problems, 5 for no good-compliance on tablets consumption) and 2 (for personal problem) were in *Chlorella* supplement group. No serious adverse reactions due to *Chlorella* supplementation were noted and the compliance was 85%. Participants were supplied with 360 tablets of placebo or *Chlorella* at 0-week and at 4-week respectively. They were also asked to return unconsumed tablets at the next visit (4- and 8-week respectively). All the participants were reinforced to regularly consume the tablets by a dietitian through every 2 week’s phone-call check during the intervention period.

### General and biochemical characteristics of study subjects

Table 1 presents general and biochemical parameters measured at baseline and after the 8-week *Chlorella* supplementation. Gender distribution and age were not significantly different between the *Chlorella* group and the placebo group. No significant differences were observed in the baseline levels or changed levels of body mass index, WHR, blood pressure, WBC count and lipid profiles between the two groups (Table 1). In addition, no significant differences were found in daily dietary intake and energy expenditure between the two groups (Table 2).

**Table 1:** General characteristics of study subjects before and after the 8-wk intervention

	***Chlorella *( n = 28)**		**Placebo ( n = 23)**	
	**0 week**	**8 week**	**0 week**	**8 week**
Male/female (n)	10/18		10/13	
Age (year)	36.3 ± 1.82		32.6 ± 1.77	
Weight (kg)	62.7 ± 1.89	62.4 ± 1.86	63.6 ± 2.63	63.0 ± 2.56
BMI(kg/m^2^)	22.8 ± 0.53	22.7 ± 0.51	22.7 ± 0.72	22.5 ± 0.69
Systolic BP (mmHg)	114.5 ± 3.29	114.1 ± 2.44	118.12 ± 2.52	117.8 ± 2.18
Diastolic BP (mmHg)	77.2 ± 2.32	77.2 ± 1.74	82.0 ± 2.07	79.0 ± 2.15
WBC (х10^3^/uL)	4.72 ± 0.16	4.96 ± 0.14	5.03 ± 0.17	5.21 ± 0.21
Triglyceride (mg/dL)	104.0 ± 11.79	114.6 ± 13.0	129.2 ± 24.2	120.4 ± 19.5
T-chol (mg/dL)	183.0 ± 6.42	177.5 ± 5.20	181.3 ± 6.65	178.7 ± 5.81
HDL-chol (mg/dL)	48.1 ± 2.75	48.6 ± 3.27	49.1 ± 2.64	51.3 ± 2.76
LDL-chol (mg/dL)	114.1 ± 6.76	106.0 ± 4.86	106.6 ± 5.46	101.7 ± 4.06

Mean ± SE or frequency.

Tested by paired t-test (within-group comparison), and by independent t-test (intergroup comparison for initial value).

There were no significant differences in ‘within-group comparison’ nor ‘intergroup-comparison’.

BMI: body mass index, BP: blood pressure, WBC: white blood cells.

**Table 2:** Daily food intake and total energy expenditure in Koreans before and after the 8-wk intervention

	***Chlorella *( n = 28)**		**Placebo ( n = 23)**	
	**0 week**	**8 week**	**0 week**	**8 week**
TEE (kcal)	2323 ± 64.5	2348 ± 60.2	2424 ± 85.0	2470 ± 82.7
Estimates of daily nutrient intakes				
TCI (kcal/d)	2284 ± 47.2	2288 ± 50.5	2221 ± 93.6	2187 ± 96.2
TEE/TCI	1.02 ± 0.02	1.03 ± 0.02	1.13 ± 0.07	1.17 ± 0.06
CHO (%)	61.6 ± 0.15	61.6 ± 0.17	61.6 ± 0.23	61.7 ± 0.24
Protein (%)	16.7 ± 0.17	16.8 ± 0.15	16.3 ± 0.17	16.6 ± 0.21
Fat (%)	22.0 ± 0.18	21.8 ± 0.17	22.3 ± 0.23	22.0 ± 0.26
Fiber	22.6 ± 1.38	22.3 ±1.31	21.8 ± 1.60	22.4 ± 1.89

Mean ± SE.

Tested by paired t-test (within-group comparison), and by independent t-test (intergroup comparison for initial value).

There were no significant differences in ‘within-group comparison’ nor ‘intergroup-comparison’.

TEE: total energy expenditure, TCI: total calorie intake, CHO: carbohydrate.

### Serum concentrations of cytokines before and after the intervention

**Figure 1:**
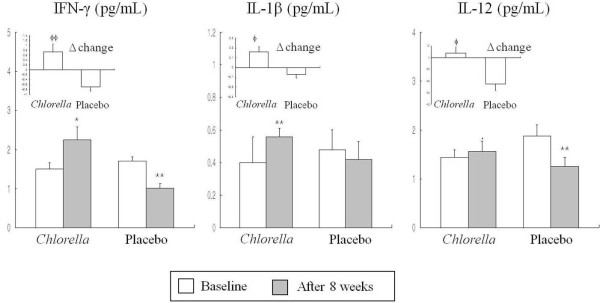
**Serum concentration of IFN-γ, IL-1β and IL-12 before and after the 8-week *Chlorella *supplementation.** Mean ± SE, tested by paired t-test (intra group comparison) or independent t-test (intergroup comparison). There were no significant differences in the baseline values between the *Chlorella* group and the placebo group. ^*^P <0.05, ^**^P <0.01 compared with the value at baseline in each group; ^ϕ^P <0.01, ^ϕϕ^P <0.001, comparison of Δ change value between the *Chlorella* group and the placebo group.

Figure 1 shows serum concentrations of INF-γ, IL-1β and IL-12 between the *Chlorella* group and the placebo group before and after the intervention. Initial levels of these cytokines were not significantly different between the two groups. After the intervention, serum concentrations of INF-γ (p<0.05) and IL-1β (p<0.001) significantly increased in the *Chlorella* group. In addition, the net change values of INF-γ (p<0.001), IL-1β (p <0.01) and IL-12 (p<0.01) before and after the intervention were significantly different between the *Chlorella* group than the placebo group.

### *Natural Killer* cell activity

**Figure 2:**
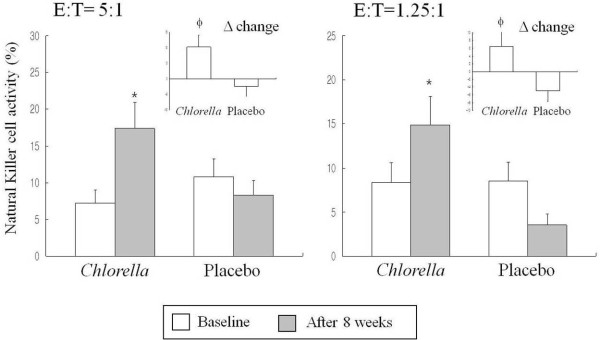
***NK *cell activity of effector cells before and after the 8-week *Chlorella *supplementation.** Mean ± SE, tested by paired t-test (intra group comparison) or independent t-test (intergroup comparison). There were no significant differences in the baseline values between the *Chlorella* group and the placebo group. ^*^P <0.05, compared with the value at baseline in each group; ^ϕ^P <0.05, comparison of Δ change value between the *Chlorella* group and the placebo group. E: effector cells (peripheral blood mononuclear cells from subjects), T: target cells (K562 cells).

*Natural Killer (NK)* cell activities (%) were measured based on the ratios of effector cells (E) (PBMC) from each participant to Target cell (T) (K562 cells) as 5:1 or 1.25:1. As shown in Figure 2, *NK* cell activities from both conditions (E:T=5:1 and 1.25:1) were significantly enhanced in the *Chlorella* group after 8 weeks (p <0.05, p<0.05, respectively). The activities seemed slightly decreased in the placebo group, but it was not statistically significant. In addition, significant differences in the net change values were observed between the *Chlorella* group than the placebo group (E:T=5:1, p<0.05, and E:T=1.25:1, p<0.05, respectively).

### Correlations among the changed levels of IL-12, IFN-γ and IL-1β in serum, and *NK* cell activity

**Figure 3:**
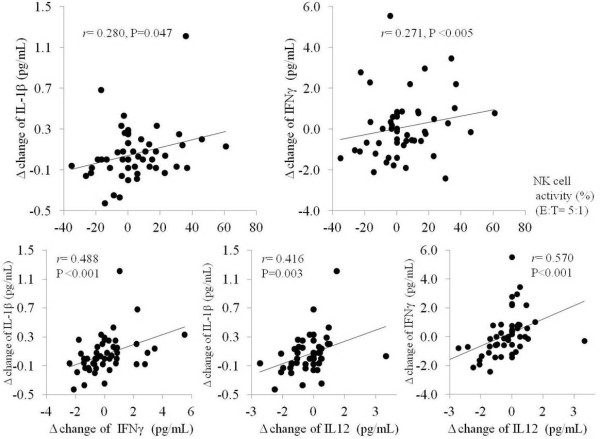
**Relationship among changed levels of *NK *cell activity and serum IFN-γ, IL-1β and IL-12. ***r* :Pearson correlation coefficient. E: effector cells (peripheral blood mononuclear cells from subjects), T: target cells (K562 cells).

As shown in Figure 3, net changes of *NK* cell activity (%) (E:T=5:1) were positively correlated with those of serum IL-1β (r=0.280, p=0.047) and IFN- γ (r=0.271, p<0.005). Signficant strong positive correlations were also observed among the changed levels of serum cytokines; betwwen IFN-γ and IL-1β (r=0.448, p<0.001), between IL-12 and IL-1β (r=0.416, p=0.003) and between IL-12 and IFN-γ (r=0.570, p<001).

## Discussion

This present study shows that 8-week supplementation of *Chlorella* tablets may give a beneficial immunostimulatory effect to normal (uninfected) people by enhancing the *NK* cell activity and producing INF-γ and IL-12 as well as IL-1β, the Th-1 cell-induced cytokines.

Helper T lymphocytes (Th) are divided into two functional subclasses, Th-1 and Th-2 cells based upon the cytokines that they produce and their effects on cell mediated and humoral immunity [28]. Th-1 cells produce IL-2, INF-γ, TNF-α and IL-12 and enhance cell-mediated immunity, thereby inhibiting cell-mediated immunologic activities. On the other hand, Th-2 cells produce IL-4, IL-5, IL-6 and IL-10, and upregulate humoral immunity. Th-1 and Th-2 derived cytokines also cross-regulate each other in various clinical conditions. Hasegawa *et al* reported in his mice model infected with *Listeria monocytogenes* that *Chlorella* extract (from *Chlorella vulgaris, CVE*) augmented IL-12 and IFN-γ [29], both of which promote the differentiation of naive CD4+ T cells into Th-1 cells producing IFN-γ, but inhibited the generation of Th-2 cells producing IL-4. In his other report, oral administration of *CVE* in mouse model inhibited immunoglobulin E (IgE) production against casein together with an impaired Th-2 response [11]. Ewart*et al* shows that *Chlorella* extract (from *Chlorella pyrenoidosa*, CPE) induces a Th-1 patterned cytokine response (IFN- γ and TNF-α) and a strong anti-inflammatory regulatory cytokines response (IL-10) in human PBMC stimulated *ex vivo*[30]. In our study, subjects who consumed *Chlorella* tablets for 8 weeks had a significant increase in serum levels of INF-γ and an increasing tendency in the levels of IL-12 compared with those who consumed placebo, which indicates that *Chlorella* supplementation may induce the production of INF- γ and IL-12 favoring a Th-1 mediated immune reaction in humans.

We also found the increased levels of serum IL-1β which is a member of the IL-1 cytokine family and produced by activated macrophages [31]. This cytokine is an important mediator of the inflammatory response, and is involved in a variety of cellular activities, including cell proliferation, differentiation, and apoptosis [31]. IL-1β stimulates multiple cells to act as immune or inflammatory response effector cells. Hunter *et al*[32] reported in his severe combined immuno-deficient mice models that IL-1β is required for IL-12 to stimulate production of IFN-γ by NK cells [32]. According to Tominaga *et al*[33], IL-12 and IL-1β synergistically induce T cells to proliferate and produce the IFN-γ and IL-12-stimulated T cells which responded to IL-18 or IL-1β by their proliferation and IFN- γ production, although the levels of IL-1β-induced responses were lower. Our result may be partially in accordance with the report of Ishikawa *et al* showing that a polysaccharide-rich fraction from *Chlorella pyrenoidosa* induced IL-1β and TNF-α in macrophage cells [34]. Taken together, *Chlorella* cells appear to contain immunostimulatory principles that stimulate a Th-1 based response. However, we did not measure the Th-2 derived cytokines such as IL-4 or IL-6, thus we may need to confirm these cytokines in further studies.

In our study, people who consumed *Chlorella* had a significant increase in *Natural Killer* (*NK*) cell activities. This result might be partly in accordance with the report of Dantas *et al* showing that oral administration of *Chlorella* extract (*CVE)* significantly increased the *NK* cell activity in normal (non-infected) mice as well as in the *Listeria monqtogenes* infected mice [35]. In addition, the *CV*-treated animals presented a dose-related increased survival rate. *NK* cells were first identified by their cytotoxtc activity against tumor cells, suggesting a role in immunological surveillance against neoplasia. Emerging evidences demonstrated that *NK* cells are important mediators of innate resistance against a variety of pathogenic intracellular microorganisms [36]. Their main function is the production of early IFN-γ, which is crucial to activate antimicrobial macrophage functions [37-42]. The kinetics of IFN-γ production by *NK* cells following infection is extremely fast, providing a source of functional cytokine at the critical time of the T-cell expansion [39,43,44]. In our study, changed level of *NK* cell activity after the intervention shows a significant positive relationship with that of serum IFN-γ. Signficanty strong positive correlations were also observed among the changed levels of IFN-γ, IL-12 and IL-1β, Th-1 patterned cytokines.

We may need to consider further study with increased number of study subjects and for longer period in the future, to confirm and clarify the result pattern. As shown in the Figures, serum levels of INF- γ and IL-12 were significantly decreased in the placebo group after 8 weeks, even though the net change of these cyotkines were significantly bigger in the *Chlorella* group than in the placebo group. We tried to search the factors which might affect the decrease of these parameters in the placebo group, but it is not easy to address the reason in a word. First, we thought the possibility of lactose, the contents in the placebo affecting cytokine levels, but it seemed not influence the cytokine levels. Second, there were no significant difference in the baseline value between the two groups, and the net change values were generally much greater in the Chlorella group than those in the placebo group, even though the direction for increment or decrement were not the same between the two groups. However, the correlation pattern among *NK* cell activity and cytokines were consistently in the positive direction, which may indicate that the measurements and the results are reliable.

## Conclusion

This study specifically focused on a normal healthy (non-infected) Koreans, so the results cannot be generalized to patients, other ethnic or geographical groups whose biochemical characteristics may differ from those in our subjects. Despite these limitations, 8-weeks of *Chlorella* intake in healthy Koreans increased the *NK* cell activity and produced INF-γ and IL-12 as well as IL-1β, the Th-1 cell-induced cytokines. In addition, changes in the *NK* cell activity positively correlated with those in the cytokines after the intervention. These results add to the growing literature on the beneficial immunostimulatory effect of *Chlorella* supplementation through a clinical human study.

## Abbreviations

BMI: Body mass index; BP: Blood presusure; E: Effector cell, INF, Interferon; IL: Interleukin; HDL-C: High density lipoprotein cholesterol; LDL-C: Low density lipoprotein cholesterol; *NK*: *Natural killer*; PBMC: Peripheral blood mononuclear cells; T: Targeted cell; TEE: Total energy expenditure; WBC: White blood cell.

## Competing interests

None of the authors have any conflicts of interest in relation to the materials presented in this paper.

## Authors’ contributions

All the authors were involved in the development of the study protocol and the experimental design. All the authors read, commented on, and contributed to the submitted manuscript. Sample collection and experiments were performed by J.H.K., S.H.B. Y.W., J.K.H., B.G.K. and O.Y.K. Data were analyzed by J.H.K. S.H.B. and O.Y.K. The manuscript was written and revised by O.Y.K. and J.H.L. O.Y.K. and J.H.L. provided the research funding. All authors read and approved the final manuscript.
